# Harnessing *Moringa oleifera* for Immune Modulation in Cancer: Molecular Mechanisms and Therapeutic Potential

**DOI:** 10.3390/ijms27010263

**Published:** 2025-12-26

**Authors:** Mounir Tilaoui, Jamal El Karroumi, Hassan Ait Mouse, Abdelmajid Zyad

**Affiliations:** 1 Laboratory of Biotechnology, Environment, Agrifood, and Health, Department of Biology, Faculty of Sciences Dhar El Mahraz, Sidi Mohamed Ben Abdellah University, Fez 30000, Morocco; 2 Laboratory of Biomolecular Chemistry, Natural Substances and Reactivity, Faculty of Sciences Semlalia, Cadi Ayyad University, Marrakech 40000, Morocco; jamalelkarroumi@gmail.com; 3 Laboratory of Agro-Industrial and Medical Biotechnology, Team of Experimental Oncology and Natural Substances, Cellular and Molecular Immuno-Pharmacology, Faculty of Sciences and Technology, Sultan Moulay Slimane University, BP 523, Beni-Mellal 23000, Morocco; aitmouse@gmail.com (H.A.M.); a.zyad@usms.ma (A.Z.)

**Keywords:** *Moringa oleifera*, chemical composition, immune system, cancer, immunomodulation, molecular mechanisms

## Abstract

*Moringa oleifera,* widely recognized as the horseradish tree or drumstick tree, is classified within the *Moringaceae* family, which comprises 13 species predominantly distributed across tropical and subtropical regions. The plant possesses a variety of therapeutic, nutritional, and beneficial health properties, including its potential to enhance the immune system. The present work provides extensive bibliographic research addressing the chemical composition of *Moringa oleifera* and its immunomodulatory properties with a focus on the cellular and molecular mechanisms involved in the regulation of immune function, which is crucial in unchecked cell proliferation and metastasis. The chemical composition of *Moringa oleifera*, including kaempferol, chlorogenic acid, quercetin, and niazimicin, varies between different biological parts of the plant (seeds, leaves, roots, and stems). The presence of these various chemical compounds contributes to the plant’s effect on the immune response via different pathways. Several studies indicate that *Moringa oleifera* mitigates inflammation by suppressing key pro-inflammatory mediators, such as TNF-α, IL-1β, inducible nitric oxide synthase (iNOS), prostaglandin E2 (PGE-2), and cyclooxygenase-2 (COX-2), while simultaneously enhancing anti-inflammatory mediators through activation of PPAR-γ. Furthermore, the immunomodulatory properties and possible application in health promotion and disease prevention, especially in cancer therapy, are discussed. Studies indicate that *Moringa oleifera* can modulate the tumor microenvironment (TME) by reducing Treg polarization, enhancing NK cell cytotoxicity, and prompting the proliferation and clonal expansion of CD8^+^ and CD4^+^ T lymphocytes. Together, *Moringa oleifera* could be considered for the treatment of conditions related to immune dysregulation, such as cancer.

## 1. Introduction

The treatment of immune-mediated inflammatory diseases, such as asthma, allergies and autoimmune diseases due to disorders of the immune system requires the modulation of the innate and adaptive immune responses [1]. Immunomodulation refers to the regulation or alteration of the immune system’s activity in response to a given internal or external stimulus, often achieved through the administration of specific compounds or drugs [2]. There are numerous chemical immunomodulators, including immunosuppressive and immunostimulatory agents such as dexamethasone, fluticasone, prednisolone, prednisone, and hydrocortisone, which have been used to treat and modulate various inflammatory diseases.

Recombinant therapeutic proteins have gained increasing importance as promising treatments for immunodeficiency, infectious diseases, and cancer [3,4,5]. Cyclosporin A is an example of a fungal cyclic peptide widely used as an immunosuppressant drug to prevent rejection in organ-transplant recipients and to treat patients with autoimmune diseases. Nevertheless, most of these conventional drugs have been associated with several adverse effects [6,7]. Duodenal mucosa and gastric mucosal epithelium damage are the commonest adverse effects caused by non-steroidal anti-inflammatory drugs [8,9,10,11]. Other side effects, notably increased skin fragility and reduced bone marrow function, have been linked to the use of immunosuppressive drugs such as corticosteroids [12,13,14,15].

Cyclosporin A is known to cause a variety of adverse metabolic side effects and toxicities, including hypomagnesemia, hypertension, hypercalciuria, hyperkalemia, hepatotoxicity, nephrotoxicity, and gingival hypertrophy [16]. Therefore, new, safer, and more effective drugs are required as alternatives to current treatments.

Natural products are still a significant source of leads for new immunomodulatory drugs. Many plants were reported to exhibit immunomodulatory activities and to modulate immune functions. The present work provides extensive bibliographic research addressing the chemical composition of *Moringa oleifera* and its effect on the immune system, exploring its cellular activities and its molecular impacts on immunoglobulins, regulatory pathways, and cytokines.

## 2. Methods/Literature Search Strategy

This comprehensive narrative review was conducted to explore the immunomodulatory and therapeutic potential of *Moringa oleifera*, especially on cancer therapy, with an emphasis on molecular mechanisms, and the phytochemical composition of its various parts (leaves, seeds, pods, flowers, and roots). Relevant studies published primarily between 2020 and 2025 were conducted across databases of PubMed/Medline, Scopus, Web of Science, Google Scholar, and Cochrane Library to identify pertinent articles. The search terms used included “*Moringa oleifera*” “chemical composition” “immune system” “immune modulation” “ant-inflammatory” “cytokines” “immunotherapy” “cancer” and “molecular mechanism”. Boolean operators (AND, OR) were used to effectively optimize the combination of these keywords for the literature research.

Studies were selected based on their relevance to preclinical, mechanistic, and clinical evidence, prioritizing peer-reviewed reports. Articles lacking methodological rigor, sufficient experimental detail, or unrelated to the topic were excluded. Although a formal risk-of-bias assessment was not conducted, priority was given to high-quality experimental and clinical studies to ensure the robustness, reliability, and validity of the synthesized findings.

## 3. Chemical Composition of *Moringa oleifera*

Each component of the plant, the leaf, root, bark, seed, flower, and pod, contains important nutritional resources [17]. Over 100 chemical constituents have been identified in *Moringa oleifera*, including proteins, fats, vitamins, minerals, carbohydrates, and dietary fibers [18]. Moreover, a multitude of phytochemicals have been identified in various parts of this plant, each offering diverse health-promoting properties [19,20].

### 3.1. Seeds

**Figure 1:**
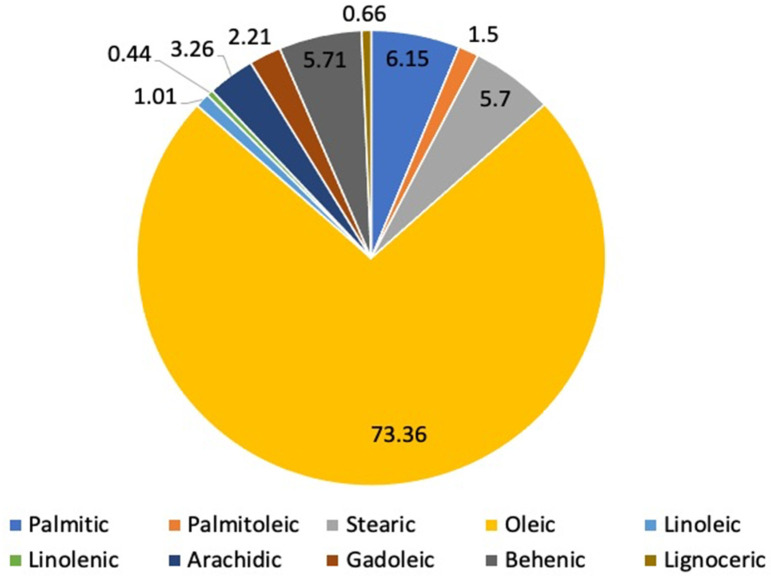
Major fatty acids identified in *Moringa oleifera* seeds.

The seeds are spherical and enclosed within a semi-permeable brown seed coat, characterized by three papery white wings. A single tree can produce approximately 15,000 to 25,000 seeds per year [21,22], each with an average mass of 0.3 g, of which about 25% is attributed to the seed coat [23]. The oil extracted from *Moringa oleifera* seeds, globally known as Ben oil, has a yield of approximately 40% [24]. According to Corbett et al., the seed oil of *Moringa oleifera* is of the oleic type with a high content of oleic acid [25]. This characteristic is particularly advantageous in the context of growing preference for replacing polyunsaturated vegetable oils with those rich in monounsaturated fatty acids, which exhibit high oxidative and thermal stability, even under high-temperature conditions such as frying. *Moringa oleifera* seed oil contains all the major fatty acids present in olive oil and is also distinguished by its notable concentration of behenic acid (C22:0), ranging from 5 to 6%. In addition, it contains small amounts of gadoleic acid (C20:1) and lignoceric acid (C24:0), along with trace levels of erucic acid (C22:1) and cerotic acid (C26:0). Figure 1 illustrates the main fatty acids found in the oil of *Moringa oleifera* seeds [26]. A study has shown that *Moringa* oil is rich in tocopherols, which are fat-soluble vitamins and powerful natural antioxidants [27].

### 3.2. Leaves

**Figure 2:**
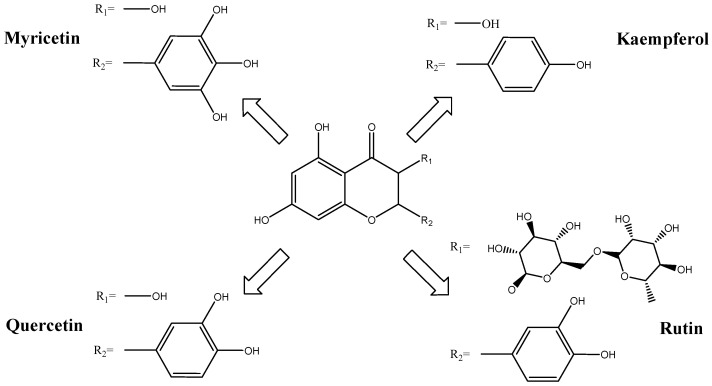
Chemical structures of representative flavonoids from *Moringa oleifera* leaves.

Nutritional analyses have shown that *Moringa oleifera* leaves are rich in vitamins, minerals, and proteins [28]. Through the process of hydrodistillation, the leaves of *Moringa oleifera* yielded a pale-yellow oil, accounting for 0.05% of the dry mass [29]. The first studies on *Moringa oleifera* date back to the 1970s. Recent comprehensive phytochemical analyses have identified more than 90 to over 100 compounds in *Moringa oleifera* leaves, including phenols, flavonoids, amino acids, and sugars, highlighting its diverse bioactive profile [20,30]. In 1982, several sugars such as L-arabinose, D-galactose, glucuronic acid, L-rhamnose, D-mannose, and D-xylose were identified in the following proportions: 14.5:11.3:3:2:1:1 [31]. Subsequently, in 1994, two nitrile glycosides, niazirin and niazirinin, were isolated by Shaheen Faizi et al. [32]. The same authors successfully identified glucosinolates, which, upon enzymatic hydrolysis by myrosianse, can produce isothiocyanates such as 4-[(α-L-rhamnosyloxy) benzyl]isothiocyanate, known for their various biological activities [33,34,35,36]. *Moringa oleifera* is known for its antioxidant activities, which are attributed to the presence of several flavonoids such as rutin, kaempferol, and quercetin [37] (Figure 2).

**Figure 3:**
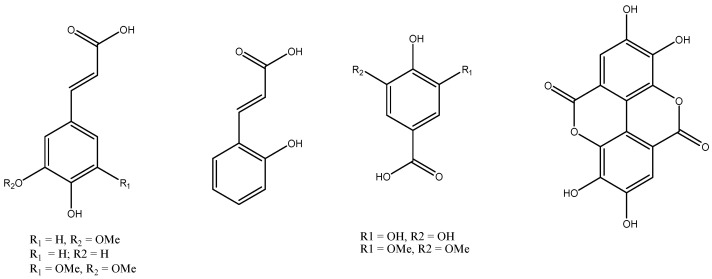
Structures of key phenolic acids present in *Moringa oleifera* leaves.

Recent investigations suggest that isothiocyanate and astragalin serve as key chemical markers for standardizing *Moringa oleifera* extracts [38], given their well-documented anti-inflammatory and antioxidant properties [39]. To quantify isothiocyanate, a specific HPLC-based cyclocondensation method was developed and applied to analyze extracts from various plant parts, such as leaves, immature seeds, mature seeds, and pods. The leaves of *Moringa oleifera* are particularly abundant in bioactive compounds, among which phenolic acids represent a prominent class [40]. Several studies have been conducted to identify and quantify these compounds with *Moringa oleifera* leaf extract. Among the most frequently reported phenolic acids are gallic acid, ferulic acid, and o-coumaric acid [41]. Figure 3 illustrates the major phenolic acids identified in *Moringa oleifera* leaves.

### 3.3. Roots

While the leaves of *Moringa oleifera* are the most commonly utilized, the roots also exhibit notable nutritional and therapeutic properties [19,42]. The root composition includes carbohydrates, proteins, minerals, and dietary fiber, along with important levels of antioxidant compounds such as phenolics and flavonoids [43]. Although these bioactive constituents are present in smaller concentrations compared to the leaves, the roots still provide essential vitamins, notably vitamin C, vitamin E, and multiple B-complex vitamins.

### 3.4. Stems

In addition to the roots, other parts of *Moringa oleifera*, including the stem, bark, and flowers, also exhibit a rich profile of bioactive phytochemicals [44]. While their chemical composition largely overlaps with that of leaves, these plant components represent significant sources of alkaloids, flavonoids, and glycosides. Moreover, compounds including steroids, terpenoids, and phenolic acids have also been identified [45]. It is worth noting that the chemical composition may be influenced by several factors, such as the plant’s geographical origin, age, genetic variability, and the extraction techniques employed [46,47,48,49].

## 4. The Impact of *Moringa oleifera* on the Immune System

### 4.1. Moringa oleifera Effect on Innate and Adaptive Immunity

*Moringa oleifera* is a widely known medicinal plant indigenous to the Indian subcontinent. It has been used for centuries in Ayurvedic medicine for its healing, nutritional, rejuvenating, and preventive properties [30,50,51]. Recent investigations have underscored its potential in modulating immune system activities [52,53,54,55,56]. The plant harbors various bioactive compounds, including flavonoids, polyphenols, and ascorbic acid, which contribute to its immunomodulatory effects [56,57].

Evidence suggests that *Moringa oleifera* extracts can increase the production of immune cells such as lymphocytes and macrophages [58,59,60,61]. This is important, as these cells play a critical role in fighting infections within the body. Notably, *Moringa oleifera* exhibits anti-inflammatory activity that reduces inflammation, thereby promoting improved immune function [62]. Overall, the research indicates that *Moringa oleifera* exerts beneficial effects on both innate and adaptive immune systems (Table 1 and Table 2). To better elucidate the mechanisms by which *Moringa oleifera* modulates immunity, we investigated the associated cellular responses and cytokine signaling in both innate and adaptive immunity.

### 4.2. Cellular Responses and Cytokine Pathways in Innate and Adaptive Immunity

Recent evidence identifies *Moringa oleifera* as a source of bioactive compounds with immunomodulatory effect [54,58,60,81]. *In vivo*, *Moringa oleifera* leaf extract enhanced natural killer cell activity and stimulated cytokine production [64], highlighting its potential to modulate innate immune surveillance with potential translational relevance for immune-based therapies.

**Figure 4:**
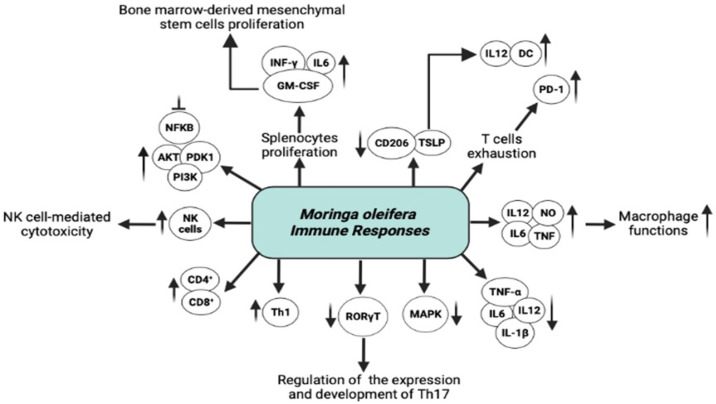
*Moringa oleifera* regulates diverse factors to modulate immune responses. *Moringa oleifera* modulates immunity through interconnected cellular and molecular pathways. Innate immunity is enhanced via neutrophile chemotaxis, macrophage phagocytic and secretory functions, NK cell-mediated cytotoxicity and the activation of bone marrow-derived mesenchymal stem cells that contribute to immune repair and cytokine balance. Upregulation of GM-CSF and NO further amplifies myeloid and effector cell activity. Adaptive immunity is regulated through CD4^+^ T-cell differentiation, balancing Treg/Th17 ratios, enhancing Th2 functions via RORγT, leading to increased IL-10 and TGF-β and reduced IL-6, IL-1beta and TNF-α. Key signaling pathways, including NF-κB, MAPK, PI3K/Akt, PDK1, STAT, TSLP, and PD-1 are modulated to maintain immune homeostasis, suppression of chronic inflammation and regulation of programmed cell death. AKT, Protein Kinase B; DC, Dendritic cell; GM-CSF, Granulocyte-Macrophage Colony-Stimulating Factor; IFN, interferon; MAPK, Mitogen-activated protein Kinase; NFκB, Nuclear factor kappa-light-chain-enhancer of activated B cells; NK, Natural killer; NO, Nitric oxide; PD-1, Programmed cell death protein 1; PDK1, 3-Phosphoinositide-dependent protein kinase-1; PI3K, Phosphoinositide 3-Kinase; RORγT, Retinoic acid receptor-related orphan receptor gamma t; STAT, Signal transducer and activator of transcription; TNF, Tumor necrosis factor; TSLP, Thymic stromal lymphopoietin. ⟶ Promote/lead to; ↑ upregulate/increase expression; ↓ downregulate/decrease expression. ⊥ inhibition.

The modulatory properties of *Moringa oleifera* on immune cell activation were confirmed by Zhuping Dong et al. [96]. The result shows through an in vitro experiment that the polysaccharide MOP-1 (glucose, galactose, and arabinose) enhances the proliferation and activation of immune cells through increasing the production of reactive oxygen species, nitric oxide, along with pro-inflammatory mediators IL-6 and TNF-α through upregulation of their mRNA expression in RAW 264.7 cells (Figure 4) [96].

The *Moringa oleifera* roots polysaccharide MRP-1 was also reported to significantly potentiate anti-inflammatory effects observed in both in vitro and in vivo models via attenuating the release of nitric oxide and iNOS mRNA expression induced by TNF-α, suggesting its potential use as a natural treatment for inflammatory diseases (Figure 4) [97]. Building on the characterization of cellular responses and cytokine pathways, we next explored how these mechanisms orchestrate multilevel modulation of innate and adaptive immunity.

### 4.3. Multilevel Modulation of Innate and Adaptive Immunity

#### 4.3.1. Gut Microbiota Modulation

*Moringa oleifera* promotes a balanced gut microbiota, enhancing immune homeostasis, reducing inflammation, and improving immune function [56]. It has also been demonstrated in animal experimental models that *Moringa oleifera* polysaccharides participate in the homeostasis of the gut microbiota by enhancing the proliferation of beneficial bacterial populations in the human gut, including *Lactobacillus* species, and a decrease in the abundance of pathogenic bacteria in C57BL/6 mice. *Moringa oleifera* polysaccharides (MOPs) modulate gut microbiota through prebiotic activity, selectively enriching beneficial bacteria, including *Bacteroides* and *Lactobacillus*, while inhibiting pathogenic bacteria, thereby enhancing microbial diversity and homeostasis [98,99,100]. Fermentation of MOPs by gut microbiota produces short-chain fatty acids (SCFAs) such as butyrate, acetate and propionate, which act via G-protein-coupled receptors (GPR41/43) to strengthen intestinal barrier integrity, regulate mucosal immunity, and suppress pro-inflammatory NF-κB signaling pathways [101,102,103]. Furthermore, MOPs influence the gut-associated lymphoid tissue (GALT), enhancing a balanced Treg/Th17 cell ratio and promoting anti-inflammatory cytokines production (e.g., IL-10, TGF-β and IL-35) while decreasing pro-inflammatory cytokines (e.g., IL-6, TNF-α) [104,105,106,107].

Together, these mechanisms form an integrated bidirectional gut-immune network, linking microbial communities to systemic immune homeostasis and highlighting the therapeutic potential of MOPs in managing inflammatory and immune-related disorders [98,106,107,108]. The composition of gut microbiota plays a central role in shaping immune responses and preserving systemic homeostasis. *Moringa oleifera* supports this balance through promoting beneficial microbes, which in turn enhances anti-inflammatory signaling and immune homeostasis.

#### 4.3.2. Immune Modulation and Anti-Inflammatory Balance

*Moringa oleifera* promotes immune homeostasis through enhancing anti-inflammatory cytokines while suppressing proinflammatory mediators. Its bioactive compounds act via key signaling pathways, including MAPK, NF-κB and PI3K/AKT/mTOR [35,109,110,111], thereby mitigating tissue damage and improving outcomes in inflammatory disorders. Additionally, *Moringa oleifera* polysaccharides were found to modulate the immune system, to increase the levels of anti-inflammatory cytokines, and reduce the inflammatory bowel disease [102]. Moreover, administration of *Moringa* leaf extract at a concentration of 100 mg/kg to mice enhances the production of eosinophil cells, immature neutrophils, and lymphocytes while reducing the amount of segmented neutrophils and monocytes [70]. Another study investigates the impact of *Moringa oleifera* leaf extract on proliferation capacity, physiological parameters, immune responses, and the expression of immunity-related genes in prawns exposed to both *Vibrio anguillarum* and ammonia stress [112]. The results showed that administration of 0.5% *Moringa oleifera* in prawns -after being exposed to ammonia stress- increases the levels of haemolymph SOD, glutathione peroxidase, NO, iNOS, and HSP70 upregulation in hepatopancreas while reducing the levels of haemolymph catalase, peroxiredoxin 5 in hepatopancreas, and nuclear factor-kappa-B-inhibitor alpha (IκB-α) expression compared to the control group. These findings indicate that *Moringa oleifera* leaf extract has a protective effect against ammonia-induced stress [112]. Similarly, Z Abidin et al. demonstrated that feeding whiteleg shrimp with 2.5 g of *Moringa oleifera* leaves’ extract significantly enhanced the innate immunity of the shrimp by increasing the production of total hemocyte count, phenoloxidase, phagocytic rate, phagocytic index, and superoxide anion. Also, the extract improved the shrimp’s resistance against *Vibrio alginolyticus*, a common pathogen in shrimp farming [113]. These findings suggest that consumption of moringa in the diet may increase the upregulation of certain genes associated with the immune system involved in catalytic activity, antimicrobial defense, antioxidant enzyme activity, and blood clotting protein production.

In agreement with the previous studies, *Moringa oleifera* leaves ethanolic extract exhibits immunomodulatory effects in broiler chicks at a dose of 200 mg/L in drinking water by increasing differential and total leukocyte counts, enhancing both the phagocytic index and phagocytic activity, elevating serum hemagglutination inhibition antibody levels against Newcastle disease virus, and reducing the mortality rate. In addition, it promotes the immune system function of the Bursa of Fabricius, spleen, and thymus indices, and stimulates lymphocytes proliferation induced by the *Moringa oleifera* leaves ethanolic extract [114]. These findings into multilevel immune regulation provide a foundation for assessing how *Moringa oleifera* engages these mechanisms to exert immunomodulatory and anti-inflammatory effects.

### 4.4. Immunomodulatory and Anti-Inflammatory Role of Moringa oleifera

*Moringa oleifera* has gained increasing attention as a bioactive plant for its promising immunomodulatory and anti-inflammatory properties [79,80,102,115,116], with accumulating evidence demonstrating its ability to regulate essential cytokine-mediated pathways [79,102,116,117,118], modulate inflammatory signaling cascades [74,102,116,119,120], restore immune homeostasis, and improve overall immune functions [50,56,82,102,107,116,121,122]. These effects have been observed across various experimental models and clinical settings [123,124,125,126,127], underscoring its promise as a natural therapeutic intervention for the prevention and management of chronic inflammatory and immune-mediated conditions.

The protective effects of *Moringa oleifera* are mediated through multiple molecular pathways. Recent studies have elucidated that its phytoconstituents exhibit multifaceted mechanisms, including the suppression of pro-inflammatory mediators such as TNF-α, IL-1β, iNOS, PGE-2, and COX-2 [76,79,111,128], alongside the activation of anti-inflammatory signaling pathways through PPAR-γ, NF-κB and MAPK [107,119,129,130,131]. Beyond innate immune regulation, *Moringa oleifera* influences adaptive immunity by enhancing antibody responses and modulation the PI3K/AKT/mTOR axis [34,81,119,132,133], an important regulator of cellular homeostasis and immune metabolism. Supported by these mechanistic observations, an important in vivo study examined the potential protective properties of *Moringa oleifera* ethanolic leaf extract (MOE) in *Oreochromis niloticus* to mitigate oxidative stress and immune dysfunction [72] caused by abamectin, an insecticide and acaricide known for its toxic activities on aquatic organisms, also shown to have immunosuppressive activity on *Oreochromis niloticus* by reducing the levels of lysozyme, nitric oxide, and IgM. The study demonstrated that the consumption of MOE dietary supplements ameliorated the adverse impact of *Agaricus blazei* Murrill (ABM), a mushroom known for its immunomodulatory activities, on lysozyme, nitric oxide, and IgM levels in the ABM + MOE-treated *Oreochromis niloticus* group [72].

The immunomodulatory effect of MOE could be attributed to cinnamic acid, naringenin, as well as additional phenolic and flavonoid compounds [72]. Importantly, administration of MOE in the treated group with ABM has been demonstrated to suppress the release of pro-inflammatory cytokines, including TNF-α and IL-1β, while upregulating the expression of genes involved in anti-inflammatory pathways, such as TGF-β and IL-10β. Additionally, *Moringa oleifera* pod extract inhibits the activation of mitogen-activated protein kinases (MAPKs) signaling cascades, thereby further contributing to the attenuation of inflammatory responses [72].

**Figure 5:**
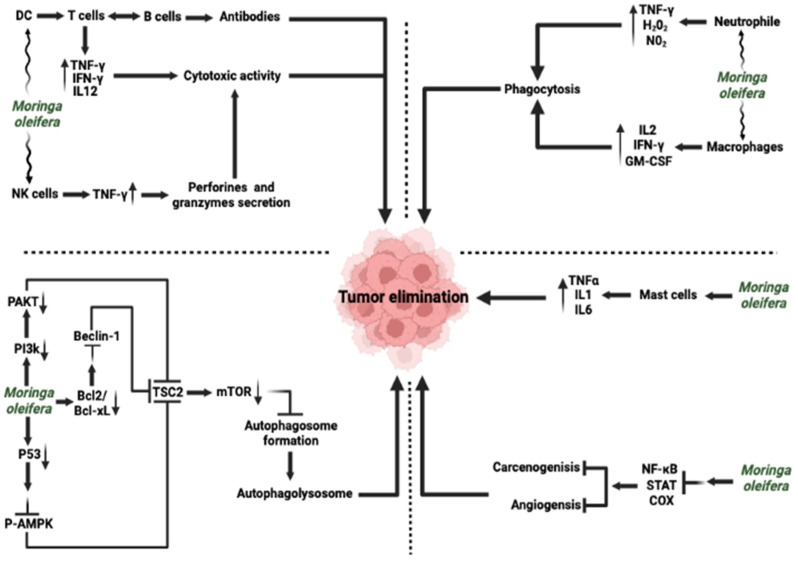
*Moringa oleifera* and its role in the modulation of cancer immunity. *Moringa oleifera* enhances innate immunity through stimulating neutrophil activation and macrophage functions, driving TNF-γ, H_2_O_2_ and NO_2_ release, and enhancing phagocytosis with IL-2, IFN-γ and GM-CSF release, thereby leading to tumor elimination. In parallel, *Moringa oleifera* modulates mast cell activity, upregulating TNF-α, IL-1 and IL-2, which contribute to antitumor responses. Furthermore, *Moringa oleifera* promotes DC maturation, T and B cell activation, contributing to antibody production, IL-12 release, and NK cell-mediated cytotoxicity via perforin and granzyme secretion. In addition, H_2_O_2_ suppresses pro-tumorigenic cyclooxygenase activity and modulate PI3K/AKT/mTOR signaling cascades through p-AKT, p-AMPK, and TSC2, and engages p53 and Beclin-1 to trigger, apoptosis, autophagolysosome through inhibition of autophagosome formation, and inhibition of carcinogenesis, with concomitant downregulation of Bcl-2 and Bcl-xL. Antioxidant and redox regulation through H_2_O_2_ and NO_2_ balance reduces oxidative stress, while modulation of NF-κB, STAT, and MAPK pathways limits chronic inflammation and supports cytotoxic T-cell and antibody-mediated responses against cancer cells. Overall, H_2_O_2_ orchestrates innate and adaptive immunity, modulates signaling pathways and balances oxidative stress to promote effective tumor elimination. COX, Cyclooxygenase; DC, Dendritic cell; GM-CSF, Granulocyte-Macrophage Colony-Stimulating Factor; IFN, interferon; mTOR, Mechanistic Target of Rapamycin; NF-κB, Nuclear factor kappa-light-chain-enhancer of activated B cells; NK, Natural killer; P-AMPK, Phosphorylated AMP-Activated protein Kinase; PAKT, Phosphorylated protein kinase B (Akt); PI3K, Phosphoinositide 3-Kinase; STAT, Signal transducer and activator of transcription; TNF, Tumor necrosis factor; TSC2, Tuberous sclerosis complex 2. ⟶ Promote/lead to; ↑ upregulate/increase expression; ↓ downregulate/decrease expression. ⊥ inhibition.

The protective effect of MOE on liver diseases [134,135,136,137] is probably due to the presence of quercetin [138], which plays a pivotal role in the prevention of liver inflammation through inhibiting NF-kB/TLR/NLRP3, inactivation of autophagy-mediated cell apoptosis (under ER stress) through the mTOR pathway [139,140,141,142,143] (Figure 5). Alterations in the mTOR signaling pathway have been associated with various human pathologies, notably liver diseases, and have also been correlated with the inhibition of cancer cell growth through suppression of the AKT/PI3K/mTOR axis [110,144] (Figure 5). Another recent study conducted by Adewale et al. demonstrates that the ethanolic extract of *Moringa oleifera* leaves exhibits an immunomodulatory effect by triggering programmed cell death pathway (apoptosis) in Jurkat cancer cells through up-regulation of CCR7, especially when those cancer cells were stimulated with CD3 and CD54 or CD28 [145]. Oral administration of aqueous extract of red *Moringa oleifera* leaves at 42 mg/kg body weight (BW) increased the number of CD4^+^CD62L^+^ and CD8^+^CD62L^+^ T cells in Salmonella typhimurium-infected BABL/c mice [145]. In a related study, the anti-inflammatory and immunostimulant effects of *Moringa oleifera* ethanolic extract were determined. The extract leads to a decrease in TNF-α on benzene-induced leukemia in Wistar rats, demonstrating its promising immunotherapy for leukemia. It has been reported that the reduction in serum levels of tumor necrosis factor alpha (TNF-α) following systemic chemotherapy in lymphoproliferative malignancies (leukemia and lymphoma) in patients is linked with response rates [146,147].

Similarly, MOEs have been shown to exert immunostimulatory effects in cyclophosphamide–induced immunosuppressed Swiss albino mice [148], supporting their potential to modulate immune responses under immunosuppressive conditions. This study found that gavage with seed and root extracts at a dose of 2 g/kg BW for seven consecutive days increased the levels of white blood cells, relative spleen and thymus weights, and enhanced immunity by supporting the proliferation and activation of lymphocytes [148]. The study indicated that the extracts decrease the delayed type of hypersensitivity response, enhance the concentration of serum IgM, and increase relative spleen weight, which was associated with a higher increase in white blood cells. Also, it was demonstrated that TNF-α and IL-2 concentrations were up-regulated (Figure 5) in the treated group with *Moringa oleifera* extracts. The study concluded that *Moringa oleifera* extracts could improve patients with common variable prognosis with immunodeficiency disorders through the stimulation of the activity of hematopoietic cells, bone marrow, lymphocytes, cellular and humoral immunity [148]. These immunomodulatory and anti-inflammatory properties form the basis for investigating the molecular mechanisms by which *Moringa oleifera* impacts cancer progression.

## 5. *Moringa oleifera* and Cancer Progression: Molecular Mechanisms and Therapeutic Potential

*Moringa oleifera* exhibits broad cytotoxic and antitumor activities through multiple mechanisms, including induction of apoptosis, cell-cycle arrest, modulation of oxidative stress via Nrf2 activation, and suppression of pro-oncogenic signaling pathways NF-κB/MAPK and inhibition of oncogenic kinases like CDK2 and Src [34,81,149,150]. Furthermore, its bioactive compounds, especially moringa isothiocyanate (MIC-1; also called moringin) and niazimicin, modulate the tumor microenvironment (TME) by increasing cytotoxic T lymphocyte and NK cell activity while decreasing Treg-mediated immunosuppression [34,110,120,151,152].

### 5.1. Apoptosis, Proliferation, and Cell-Cycle Control in Moringa oleifera-Mediated Cancer Progression

*Moringa oleifera* extract from leaves, seeds, and oils along with the purified MIC-1 have been shown to reduce tumor cell viability and clonogenicity through triggering caspase-dependent apoptosis and arresting cells at the G1/S checkpoint. In renal cancer cells, MIC-1 suppresses growth and migration by inhibiting PTP1B-dependent Src/Ras/Raf/ERK signaling, enhances the pro-apoptotic Bax/Bcl-2 ratio in xenograft models while inhibiting ERK phosphorylation (Figure 5), establishing a mechanistic bridge between upstream phosphatase targeting and downstream caspase-driven apoptosis [34]. Furthermore, phenolic fractions from *Moringa oleifera* leaves induce activation of caspases 3/7/9, alongside mitochondrial ROS production and AIF translocation in melanoma cancer cells, suggesting simultaneous caspase-dependent and independent apoptosis signaling pathways [153].

In liver cancer, more particularly hepatocellular carcinoma (HepG2), phenolic compounds from *Moringa oleifera* fruit and leaves initiate ROS-mediated cellular signaling cascade, trigger caspase-3 activity and drive mitochondrial pathway-dependent apoptosis [152]. Concurrently, another study combining in-silico CDK2 docking with in vitro validation in ER-positive breast cancer reported that *Moringa oleifera* phytochemicals, including chlorogenic acid, niazirin, kaempferol, quercetin and ellagic acid act as putative CDK2 inhibitors, supporting the inhibition of cell growth through promoting cell cycle arrest at G1/S in hormone-receptor-positive disease [154]. Beyond its role in epithelial cancers, *Moringa oleifera* leaf protein decreases proliferation, induces apoptosis and promotes cell-cycle arrest in Jurkat T-ALL cells via MAPK/AKT pathway modulation (Figure 5), underscoring its relevance in hematologic malignancies [81].

The antiproliferative and pro-apoptotic signature is consistently observed across additional cancer cell lines, with A549 (NSCLC) cells displaying caspase 9/3 activation and Bax/Bcl-2 shifts in response to *Moringa oleifera*-based extracts [155]. Similarly, colon cancer cells respond to glucosinolate-rich lead fractions with decreased proliferation [35,156], and Dalton’s lymphoma cells demonstrate both in vitro and in vivo growth inhibition with upregulated Bax, cytochrome-c release and cleaved caspase 3 along with decreased Bcl-2 expression, accompanied by cell cycle arrest at G2/M, apoptosis induction, and importantly, significant improvement in hematological parameters in mice bearing Dalton’s Lymphoma tumors [157]. Collectively, these studies converge on (1) caspase cascade engagement, (2) suppression of pro-survival signals such as ERK, and (3) CDK-axis inhibition at G1/S, providing a mechanistic basis for *Moringa oleifera* constituents as adjuncts to cytotoxic antitumor or targeted therapies, while emphasizing the need for standardized preparations to ensure reproducibility across experimental models.

### 5.2. Moringa oleifera in Cancer: Dual Regulation of Oncogenic Signaling and Oxidative Stress

Recent investigations have highlighted the dual modulatory effects of *Moringa oleifera* constituents on oncologic signaling and redox homeostasis, elucidating their potential as adjuncts in cancer therapy. Notably, the MIC-1 has been shown to inhibit migration and proliferation in renal cancer cells through modulation of PTP1B-dependent Src/Ras/Raf/ERK signaling [34]. This inhibition leads to reduced phosphorylation of Src (Tyr416), K-Rad, Raf and ERK1/2 [34], thus impairing tumor cell growth and migration. Concurrently, MIC-1 triggers the Nrf2/ARE pathway, upregulating the expression of cytoprotective genes such as NQO1 and enhancing cellular defense against oxidative stress [34]. This dual activity may potentially modulate tumor cell adaptation under stress conditions. In addition, in vivo studies further support these findings, showing that MIC-1 treatment results in increased Bax/Bcl-2 ratios in tumor tissues, a pivotal indicator of the induction of apoptosis [34]. Also, it has been reported that *Moringa oleifera* extracts modulate key oncogenic and stress-related signaling pathways implicated in multiple cancer types, such as breast, hepatocellular, melanoma, colon, NSCLC (Non-Small Cell Lung Cancer) and hematologic malignancies. These effects are mediated through the coordinated regulation of Nrf2-Keap1, TLR4/NF-κB and Wnt/β-catenin, thereby orchestrating oxidative stress and inflammatory responses, processes that are crucial to tumor growth, survival and therapy resistance [152].

### 5.3. Inhibition of Angiogenesis and Metastasis Progression

Recent studies have elucidated the potential role of *Moringa oleifera* extracts to modulate angiogenesis and metastasis processes (Figure 5), which are essential for tumor progression [158,159,160]. Although direct evidence of anti-angiogenic effect remains limited, several in vitro and in vivo investigations have demonstrated inhibition of cell migration and downregulation of metastasis-associated pathways. In a recent study, Raju and Kei (2022) reported that *Moringa oleifera* leaf extract exhibits significant anti-angiogenic activity, with the 100% aqueous extract showing an 81.33% reduction in vascular formation in the chick chorioallantoic membrane assay [161], suggesting the presence of bioactive compounds capable of inhibiting angiogenesis, a key process in tumor growth and metastasis. Furthermore, studies on *Moringa oleifera*-silver nanoparticles (MO-AgNPs) have been shown to inhibit metastasis through modulating pathways involving Snail and TGF-β. These findings suggest that MO-AgNPs may serve as a promising and effective targeted strategy to inhibit tumor metastasis [162].

Emerging evidence suggests that *Moringa oleifera* possesses significant capacity to modulate immune-regulatory networks within the TME. Notably, a recent study demonstrated that *Moringa oleifera* leaf protein inhibited the proliferation of T-lymphoblastic leukemia cells by modulating immune-related signaling pathways, specifically the MAPK (p38 and ERK) and AKT axis (Figure 5), while simultaneously inducing apoptosis, cell-cycle arrest, and autophagy [81]. Additionally, recent investigations have further revealed the ability of *Moringa oleifera*-derived polysaccharides to reprogram the TME through targeting innate immune cells. In a Lewis lung carcinoma model, *Moringa oleifera* leaf polysaccharides were shown to remodel tumor-associated macrophages from a pro-tumoral M2 phenotype toward an antitumoral M1 phenotype. This repolarization was mediated via TLR4 signaling, leading to upregulated chemokine expression CXCL9 and CXCL10 and increased intra-tumoral T-cell infiltration, thereby exerting powerful immune-activating effects [58]. In parallel, *Moringa oleifera* seed oil extract has demonstrated antioxidant, anti-inflammatory, and antitumor activities in Ehrlich ascites carcinoma-bearing Swiss albino mice treated at a dose of 500 mg/Kg [80], where treatment improved systemic oxidative stress markers through reducing malondialdehyde levels while elevating the activities of essential antioxidant enzymes, superoxide dismutase and catalase, indicating significant oxidative homeostasis amelioration. Furthermore, *Moringa oleifera* seed oil extract attenuated proinflammatory responses in tumor-bearing mice; collectively, these findings underscore its therapeutic potential to restore redox balance and mitigate the inflammatory reactions within the TME [80]. Nevertheless, caution must be taken regarding the use of *Moringa oleifera* preparations in combination with standard chemotherapy regimens, as it has been shown that in an obesity-associated triple-negative breast cancer model (MDA-MB-231 xenografts in C57BL/6J), the concomitant oral administration of *Moringa oleifera* seed extract concentrate (high-fat diet supplemented with 0.6% *w*/*w Moringa* concentrate) with conventional chemotherapy drug—comprising doxorubicin (2 mg/kg) and cyclophosphamide (100 mg/kg)—not only negated its angiogenesis-reducing benefit but also aggravated tumor progression, underscoring possible context-specific pro-tumor effects or adverse reactions [163]. These outcomes highlight the need for mechanistic and pharmacokinetic/pharmacodynamic clarity when exploring *Moringa oleifera* in combination with chemotherapy.

### 5.4. From Bench to Bedside: Addressing Translation Challenges in Moringa oleifera-Based Cancer Therapy

To advance *Moringa oleifera* as a reliable adjunct in cancer therapy, critical translational gaps must first be addressed. (i) Standardization and characterization of *Moringa oleifera* preparations. Defining Good Manufacturing Practice (GMP)-grade materials with rigorously quantified active constituents such as MIC-1, niazimicin, and principal flavonoids, alongside comprehensive impurity profiling and batch-level certificates to ensure validity and reproducibility in both preclinical and clinical investigations. Recent studies have highlighted variability in bioactive compounds across different *Moringa oleifera* sources, emphasizing the urgent need for standardized preparations [152]. (ii) A thorough understanding of pharmacodynamic, pharmacokinetic and target engagement is required. Determining human-relevant exposure levels of MIC-1 (both free and conjugated forms), evaluating tissue distribution, and confirming interaction with key molecular targets, including PTP1B and Nrf2, are crucial for predicting therapeutic outcomes and clinical efficacy [164,165,166]. Computational and experimental studies indicate that *Moringa oleifera* phytochemicals may modulate oncogenic and pro-survival proteins, such as Bcl-2 [81,144,167,168,169,170], providing mechanistic avenues that support translational potential. (iii) Context specificity should be rigorously addressed. The effect of *Moringa oleifera* can vary depending on tumor subtype, metabolic status (e.g., obesity), and concomitant treatments. Preclinical evidence demonstrates that *Moringa oleifera* modulates important signaling pathways, including YAP/TAZ, Wnt/β-catenin, Nrf2-Keap1 and TLR4/NF-κB, which play divergent roles depending on the tumor cellular context, microenvironment, and cancer-related immune-metabolic axis [152]. Consequently, tailoring interventions to the specific disease context is crucial to minimize adverse and prevent counterproductive outcomes, thereby enhancing therapeutic efficacy and advancing the development of targeted cancer therapies. (iv) Assessment of immuno-oncology endpoints should extend beyond conventional chemotherapeutic regimens. Evaluating *Moringa oleifera*’s impact on the TME, including Treg/CD8^+^ balance, NK activity and M1/M2 macrophage polarization in immunocompetent models, with or without checkpoint blockade, is essential to define its immunomodulatory potential. Emerging evidence shows that *Moringa oleifera* extracts can enhance immune activation, suggesting promising opportunities for combination with immunotherapeutic strategies [81]. (v) Early-phase clinical studies are essential to translate *Moringa oleifera* findings to humans. Phase 0 or early pharmacokinetic trials, using biomarkers such as NQO1 activation, ERK signaling and cytokine profiles can guide dosing, pharmacodynamic response, and safety before disease-focused randomized controlled trials. Despite limited clinical data on cancer research, pharmacokinetics, antioxidant and anti-inflammatory activities provide a strong rationale for systemic investigation in oncology [20].

Together, addressing these translational priorities, including standardized *Moringa oleifera* preparations, rigorous pharmacological characterization, context-specific evaluation, and early clinical investigation, will enable the rational incorporation of *Moringa oleifera* into cancer therapy while ensuring mechanistic understanding, reproducibility, and safety.

## 6. Clinical Trials on the Immunomodulatory Properties of *Moringa oleifera* in Disease Prevention and Cancer Therapy

*Moringa oleifera* has emerged as a promising candidate due to its rich phytochemical composition and demonstrated ability to modulate both innate and adaptive immune responses through interconnected mechanisms. Recent clinical studies have begun to provide evidence supporting its immunostimulatory effects in humans, reflected by improved immune biomarkers, hematological parameters, and disease-specific outcomes [123,124,125,126,127]. These trials provide a translational perspective, bridging preclinical research findings with potential clinical applications for immune enhancement, disease prevention and management. Extending on its systemic immunomodulatory properties, clinical studies have demonstrated that *Moringa oleifera* improved immune responses in patients with gum disease [171] (CTRI/2022/02/040594), increases CD4 levels and nutritional status in HIV-positive patients [127,172], and improves immunological and hematological parameters in patients undergoing highly active antiretroviral therapy [173]. Moreover, clinical investigations have highlighted the potential of *Moringa oleifera* as an adjunct in cancer immunotherapy. In particular, an aqueous leaf extract exhibited significant anticancer activities by inducing apoptosis and inhibiting tumor progression without affecting normal physiological functions [174].

Another important study showed that *Moringa oleifera* leaf polysaccharides possess the ability to modulate the TME through inducing a phenotypic switch from immunosuppressive M2 macrophages to pro-inflammatory M1 phenotypes via TLR4 targeting [58], suggesting their promising role in cancer immunomodulation. In addition, the combination of *Moringa oleifera* with other phytochemicals, including saffron or green tea, has been demonstrated to enhance both anticancer efficacy and immunomodulatory activity, highlighting the therapeutic potential of synergistic strategies in cancer prevention and therapy [54].

Overall, these clinical findings underscore the translational potential of *Moringa oleifera* for disease prevention, immune modulation, and as an adjunct in cancer immunotherapy. While clinical trials demonstrate the therapeutic benefits of *Moringa oleifera*, investigating its long-term safety and toxicity remains critical to support its sustained therapeutic application.

## 7. Long-Term Safety and Toxicity of *Moringa oleifera*

The long-term safety and toxicity profile of *Moringa oleifera* has been extensively investigated in both preclinical and clinical studies, with most evidence supporting its well-established tolerability. A consolidated overview of these findings is presented in Table 3, which summarizes the available evidence across plant parts, experimental models, dosing regimens, and key safety outcomes.

Preclinical studies on animal models have provided insights into the safety profile of *Moringa oleifera*. For instance, a 13-week repeated-dose study of an optimized aqueous leaf extract revealed no serious toxicity but recommended caution with long-term doses above 500 mg/kg in rodents, establishing a no-observed-adverse-effect level (NOAEL) close to 500 mg/kg [180]. In parallel, a 28-day repeated-dose toxicity study in rats administered orally with aqueous leaf extract exhibited no mortality or organ toxicity at 100–500 mg/kg/day, although a minor reduction in body weight was observed at the highest doses, findings that align with an acute safety margin up to 2000 mg/kg [181,182]. Moreover, disease-model studies, notably in gout, also produced favorable outcomes, further strengthening the evidence for systemic safety [183]. Reproductive and developmental toxicity assessments likewise have shown an absence of adverse effects at nutritionally relevant doses, thereby supporting its long-term safety [184,185,186]. Evidence on *Moringa oleifera* seeds and roots suggests a more restricted safety compared to leaves. A 14-day oral toxicity evaluation of a hydro-alcoholic seed extract did not report any significant toxicological effects, supporting its short-term tolerability [62,175]. In contrast, seed oil administered at 200–800 mg/kg in murine malaria models not only reduced parasitemia but also induced renal and hepatic lesions at higher doses, indicating a narrower therapeutic window for seed oils compared to *Moringa oleifera* leaf preparations [187]. Likewise, a 21-day oral administration of ethanol extract (150–600 mg/kg/day) in rats showed no nephrotoxic or hepatotoxicity on standard biochemical parameters, although the authors recommended longer-term evaluations due to the generally higher caution warranted for roots compared with leaves [188]. These findings emphasize the critical role of plant-part specificity, with leaves demonstrating broadly safer, whereas seed and root derivatives may require more cautious dosing and further long-term evaluation. Additional preclinical studies on related species, including *Moringa stenopetala* herbal tea blends, also support low toxicity in rats [189], though direct extrapolation to *Moringa oleifera* should be approached cautiously.

Clinical trials further corroborate these findings, indicating that oral supplementation with whole leaf powders or extracts is generally safe, well-tolerated and without consistent adverse effects in humans, while highlighting the need for standardized products and longer follow-up [62,190]. Both randomized controlled and observational studies have consistently reported no significant adverse effects, even at relatively high doses over extended periods. These observations are further supported by systematic reviews indicating that *Moringa oleifera* is well-tolerated, does not produce significant adverse effects, and serious toxicity [190,191,192,193]. Importantly, *Moringa oleifera* supplementation did not compromise hematological, renal, or hepatic functions, highlighting its favorable systemic safety profile [194,195,196]. Nevertheless, caution is warranted with *Moringa oleifera* parts consumption, especially the bark and roots. Overuse of these parts may carry toxicity risk due to the presence of potentially toxic bioactive compounds such as alkaloids [197,198,199,200]. Moreover, pharmacokinetic evidence indicates the possible interaction of *Moringa oleifera* with drugs metabolized by cytochrome P450 enzymes, including antihyperglycemic drugs, necessitating the need for careful use in patients receiving such therapies [192]. Rare clinical case reports have identified cutaneous reactions and potential renal/hepatic perturbations, as well as possible drug interactions, emphasizing the necessity for cautious dosing and pharmacovigilance, particularly in vulnerable populations [201]. Taken together, although *Moringa oleifera* demonstrates a generally favorable safety profile in both preclinical and clinical studies, its long-term use should be approached with consideration of potential drug–nutrient interactions, doses, and the specific parts of the plant employed.

## 8. Conclusions and Future Directions

*Moringa oleifera* has long been known to exhibit medicinal properties, and recent scientific investigations have shown its efficacy in modulating the immune system. These research findings suggest that *Moringa oleifera* plays an essential role in maintaining homeostasis between pro-inflammatory and anti-inflammatory cytokine responses and regulating the functions of CD4^+^ T-cells subsets by targeting transcription factors and their signal transducers. These results highlight its promise in managing T cell-mediated autoimmune disorders and coordinating innate and adaptive immune responses. Its extracts also enhance hematopoietic activity and strengthen both cellular and humoral immunity, suggesting benefits for immunodeficiency disorders, while supporting gut microbial balance to reinforce immune homeostasis and anti-inflammatory signaling. Beyond immune regulation, *Moringa oleifera* exhibits potent anticancer activity through inducing apoptosis, cell-cycle arrest, activation of antioxidant pathways such as Nrf2, and suppressing oncogenic signaling. Evidence also indicates its capacity to inhibit angiogenesis, metastasis, and modulate immune networks within the tumor microenvironment.

To meet accepted standards, it is important to conduct high-throughput screening, analyses of the bioactive compounds, and identify the chemical targets when studying the immunomodulatory and anti-cancer activities of *Moringa oleifera* extracts. Those extracts should be subject to qualitative and quantitative analysis through validated analytical procedures to define phytochemical levels in *Moringa oleifera* extracts.

Several bioactive compounds from *Moringa oleifera*, such as isothiocyanate, flavonoids and polysaccharides (e.g., MOP-2, MRP-1) have been shown to modulate immune signaling pathways and exhibit immunomodulatory and anti-cancer effects. Nevertheless, it is necessary to conduct further mechanistic investigations with a comprehensive update on the current state of research, through multi-scale approaches spanning cellular and molecular mechanisms, host–microbiome interactions, and systemic physiology, to better understand how these compounds regulate innate, adaptive immune system responses and tumor biology. A deeper understanding of how *Moringa oleifera* modulates λδ T cells, natural killer T cells, mucosal-associated invariant T cells, and their roles in immune-regulatory circuits within the TME will be critical to advance *Moringa oleifera* from traditional medicine toward evidence-based immunotherapy and cancer interventions.

Despite the promising therapeutic effects of *Moringa oleifera*, critical gaps remain regarding its long-term safety, potential drug–nutrient interactions, optimal dosing, and the specific plant parts employed. Accordingly, rigorous standardized preclinical testing, encompassing toxicological, pharmacokinetic, and bioavailability studies, will be essential to ensure both safety and effective translation into clinical use.

## Figures and Tables

**Table 1:** Effect of *Moringa oleifera* on innate immunity.

Plant Part Used	Form of Application	Model/System	Key Mechanistic Insights	Ref
Leaves	Leaf polysaccharide fraction	Lewis lung carcinoma (C57BL/6 mice, oral). In vitro bone marrow-derived macrophages	Activation of macrophage through toll-like receptor reprograms TAMs from M2 to M1 through TLR4, ↑ CXCL9/10, ↑ T-cell infiltration	[58,63]
Leaves	Oral administration	Rabbits exposed to heat stress	Enhancement of NK cell activity through up-regulation of perforin/granzyme secretion	[64,65,66]
Leaves	Purified protein fraction	Murine BMSCs (In vitro), followed by allogenic MLR and in vivo IgE measurement in mice	Activation and maturation of DCs via upregulation of co-stimulatory molecules and the secretion of cytokines and chemokines: ↑ CD80/CD86/MHCII; ↑ IL12 and TNF-α secretion; OX40L-TIM-4-CCL17/22 axis drives Th2 polarization; enhanced T-cell proliferation	[60,67,68]
Leaves	Oral administration	Diabetic-induced damage in male Wistar rats	Reduction in pro-inflammatory cytokines (TNF-α, IL6) and oxidative stress	[68]
Leaves	Topical ethanolic extract (10%) Moringa tea (oral drinking water)	Wistar rats with experimental burn wounds Restraint-stressed mice	Modulation of neutrophil migration/chemotaxis and enhancing phagocytosis	[59,69]
Leaves	Oral administration of ethanolic leaf extract at 10, 30 and 100 mg/Kg for 7 days	*Staphylococcus aureus*-challenged mice (mouse peritoneal macrophage)	↑ Macrophage phagocytic activity and capacity; ↑ Neutrophile percentage	[70]
Leaves	Purified polysaccharide fraction	RAW 264.7 macrophages (in vitro)	Enhances the gene expression of antimicrobial peptides such as Mop3 through ↑ pinocytosis; ↑ ROS/NO; ↑ IL6; TNF-α and iNOS expression	[71]
Pods	Boiled extract	RAW 264.7 murine macrophage cell line	Inhibits pro-inflammatory cytokines (IL6, TNF-α); suppresses iNOS and COX-2 expression; decreases NO production	[72]
Leaves	Ethyl acetate fraction	LPS-stimulated RAW 264.7 macrophages	Regulation of genes encoding pro-inflammatory cytokines through the regulation of MAPK and NF-κB signaling cascades	[73]
Seeds	Purified peptide (sequence KETTTIVR) administered orally	Dextran Sulfate Sodium-induced colitis in mice	Modulates gut microbiota and metabolomic profiles, inhibits JAK-STAT signaling, and protects the intestinal barrier	[74]
Leaves	Ethanolic extract; oral and in vitro exposure	Swiss albino mice peritoneal macrophages; RAW 264.7	Increase phagocytosis activity through activation of phagocytic receptors and engulfment efficiency	[70]
Roots	Hot water extract, ethanolic extract	LPS-induced RAW 264.7 macrophages murine macrophage cell line	Inhibits NO and TNF-α production; decreases iNOS mRNA production	[75]
Pods	Freeze-dried pod polyphenol extract	RAW 264.7 macrophages (LPS-stimulation)	↓ NO, ↓ TNF-α, strong anti-inflammatory effect	[76]
Leaves	Ethyl acetate extract (oral and in vitro)	RAW 264.7 macrophages	Inhibits NF-κB, suppresses pro-inflammatory cytokines (TNF-α, IL6); ↓ NO production	[77]
Pods	Digested boiled pod extract	Caco-2 cells (intestinal epithelial)	Suppressed inflammatory mediators; promoted epithelial anti-inflammatory defense	[78]
Leaves (aqueous and ethanolic extracts)	Oral administration and in vitro exposure	Various animal models (Wistar rats and BALB/c mice) and the RAW 264.7 macrophage cell line	Anti-inflammatory and antioxidant activities via free radical scavenging and inhibition of pro-inflammatory cytokines such as IL-1β, IL-6 and TNF-α	[68,77,79,80]

BMSCs, Bone marrow-derived mesenchymal stem cells; CCL, C-C motif chemokine ligand; CXCL, C-X-C motif chemokine ligand; DCs, Dendritic cells; iNOS, Inducible nitric oxide synthase; JAK, Janus kinase; LPS, Lipopolysaccharide; MAPK, Mitogen-activated protein Kinase; NF-κB, Nuclear factor kappa-light-chain-enhancer of activated B cells; OX40, OX40 ligand; STAT, Signal transducer and activator of transcription; TIM, T cell immunoglobulin and mucin domain containing protein 4. ↑ upregulate/increase expression; ↓ downregulate/decrease expression.

**Table 2:** Effect of *Moringa oleifera* on adaptive immunity.

Plant Part Used	Form of Application	Model/System	Key Mechanistic Insights	Ref
Leaves	Purified leaf protein	In vitro, Human T-lymphoblastic leukemia (Jurkat cells)	Modulation of T cell activation and proliferation through the regulation of T cell receptor signaling cascades	[60,81,82]
Leaves	Aqueous extract	In vitro, murine splenocyte culture	B-cell activation	[73]
Leaves	Methanolic extract	SRBC immunized Wistar rats	Antibody production via B cell receptor stimulation	[83,84,85]
Leaves	Ethanolic extract	Breast cancer cells (MD-MA-231)	↓ of NF-κB and p65; Transcription factor modulation; ↓ cancer cell viability	[86]
Leaves	Topical application	Atopic dermatitis mouse model	Promote T-helper cell differentiation through modulation of Th1, Th2, Th17 and Treg cells responses	[61,67,87,88,89]
Leaves Leaf proteins	Oral administration Protein extract	Rabbits exposed to heat stress Murine BMDCs	Induction of Foxp3 expression and promotion of Treg cells	[60,64]
Pods	Pod meal (dietary inclusion)	Broiler chickens	Enhanced growth performance; improved cell-mediated immunity	[90]
Leaves	Leaf protein fraction or extract	BALB/c mice (intraperitoneal sensitization + oral exposure, allergy model); BMDCs (in vitro) and IgE induction after DCs transfer (in vivo)	Regulation of antigen presentation and processing	[60,88,91]
Leaves	Methanolic extract	Wistar albino rats	Stimulates neutrophil and lymphocyte function; Enhances humoral immunity; increases antigen-specific antibody secretion and WBC levels	[85]
Seeds	MicroRNA-enriched extract	Human PBMCs (HIV^+^)	Modulates T-cell differentiation and memory T-cell subsets; reduces HIV replication	[92]
Leaves	Protein extract (oral and in vitro)	BALB/c mice; Murine BMDCs	Induces IgE production and Th2 polarization; activates DC; modulates humoral immunity by enhancing the production of antigen-specific antibodies	[60,93,94,95]

BMDCs, Bone marrow-derived dendritic cells; DCs, Dendritic cells; Foxp3, Forkhead box P3 gene; PBMCs, Peripheral blood mononuclear cells; SRBC, Sheep red blood cells; WBC, White blood cells. ↓ downregulate/decrease expression.

**Table 3:** Long-term safety and toxicity profile of *Moringa oleifera*: insights from preclinical and clinical studies.

Plant Part	Study Model	Dose/Duration	Key Findings/Safety Outcomes	Ref
Seeds (hydroalcoholic extract)	Rats (14-day toxicity study)	100–2000 mg/kg/day	No significant toxicological effects; normal hematological and biochemical parameters	[175]
Leaf capsules in T2DM patients	Humans: adults with T2DM (therapy naïve)	Nutritional dose, 4 weeks	Well tolerated; no hypoglycemia; renal (BUN, creatinine) and hepatic (AST, ALT) markers remained normal	[176]
Leaves (hydroethanolic extract)	Female ICR mice	Acute: 2000 mg/kg, single dose. Sub-acute: 125–1000 mg/kg daily for 28 days.	Acute: LD_50_ > 2000 mg/kg; signs of liver and kidney damage (increased AST, CK and creatinine; hepatic degeneration; renal necrosis). Sub-acute: moderate hepato-nephrotoxicity (hepatic and renal necrosis, sinusoidal dilatation; glomerulonephritis). Lower doses (125–500 mg/kg): relatively safe with minimal adverse effects.	[177]
Leaf powder	Humans: adults	400 mg capsules, 6x daily (2.4 g/day); 12 weeks	Well tolerated; no adverse effects; kidney parameters (creatinine, urea), liver enzymes (AST, ALT), and hematological markers remained normal; decreased inflammatory parameters (CRP) and improved lipid profile (LDL-C, total cholesterol)	[124]
Leaf powder (added to RUSF)	Humans: children < 5 years with moderate malnutrition	Daily supplementation: 5 weeks for the *Moringa* group and 4 weeks for the placebo group.	No renal/hepatic toxicity reported; well tolerated	[178]
Leaf powder (10% fortified porridge)	Humans (observational study): children with cerebral palsy	Daily intake (3 months)	No serious adverse reactions or increased morbidity; improved nutritional (vitamin A and protein status) and immune markers	[179]

ALT, Alanine aminotransferase; AST, Aspartate aminotransferase; BUN, Blood urea nitrogen; CK, Creatine Kinase; CRP, C-reactive protein; ICR mice, Institute of Cancer Research (mouse strain); LDL-C, Low-density lipoprotein cholesterol; RUSF, Ready-to-use supplementary food; T2DM, Type 2 Diabetes Mellitus.

## Data Availability

No new data were created or analyzed in this study. Data sharing is not applicable to this article.

## Abbreviations

The following abbreviations are used in this manuscript:

CCR7	C-C Chemokine Receptor Type 7
ER	Endoplasmic Reticulum
HSP70	Heat Shock Protein 70
iNOS	Inducible Nitric Oxide Synthase
MOP	*Moringa oleifera* Polysaccharide
Mop3	Motif 3 Protein
MRP-1	Moringa Root Polysaccharide 1
NLRP3	NOD (Nucleotide-binding Oligomerization) LRR (Leucine-Rich Repeat) Pyrin Domain-containing Protein 3
NQO1	NAD(P)H: Quinone Oxidoreductase 1
PGE-2	Prostaglandin E2
PTP1B	Protein Tyrosine Phosphatase 1B
SOD	Superoxide Dismutase
TLR	Toll-Like Receptor
TME	Tumor Microenvironment
